# Changing Diagnostic Methods and Increased Detection of Verotoxigenic *Escherichia coli*, Ireland

**DOI:** 10.3201/eid2209.160477

**Published:** 2016-09

**Authors:** Thomas Rice, Noreen Quinn, Roy D. Sleator, Brigid Lucey

**Affiliations:** Cork Institute of Technology School of Science and Informatics, Bishopstown, Cork, Ireland

**Keywords:** verotoxigenic, Escherichia coli, VTEC, molecular, detection, notifications, bacteria

## Abstract

The recent paradigm shift in infectious disease diagnosis from culture-based to molecular-based approaches is exemplified in the findings of a national study assessing the detection of verotoxigenic *Escherichia coli* infections in Ireland. The methodologic changes have been accompanied by a dramatic increase in detections of non-O157 verotoxigenic *E. coli* serotypes.

Verotoxigenic *Escherichia coli* (VTEC) can cause severe disease in humans, with signs and symptoms including diarrhea, hemorrhagic colitis, and hemolytic uremic syndrome (*1*). The primary reservoir of VTEC is ruminants, and sporadic outbreaks are commonly associated with animal contact, exposure to animal feces, food and water contamination, and person-to-person transmission (*2*).

In Ireland, a country with a population of ≈4.6 million, all VTEC infections have been notifiable to the Medical Officer of Health since 2004. Initially, these infections were included under specified infections caused by enterohemorrhagic *E. coli* (EHEC), comprising any strains of serogroups O157, O26, O103, O111, and O145 (*3*); since an amendment of September 2011, these infections have been specified as VTEC infections (*4*). Data on VTEC infections in Europe have been provided since 2008 from the member states to the European Centre for Disease Prevention and Control. These data indicate that Ireland had the highest annual incidence of VTEC infection during 2009–2014, with the exception of 2011, when a large outbreak of *E. coli* O104:H4 infections occurred in Germany (*5*). The Health Protection and Surveillance Centre releases annual reports on the number of VTEC infection cases in Ireland, which document an increase in the number of VTEC infections in Ireland during 2010–2014 (Figure). This trend is thought to be largely attributable to an increase in non-O157 VTEC infections. A low infectious dose of ≈10 microorganisms has been described as sufficient to cause disease that can lead to hemolytic uremic syndrome (*6*). Real-time PCR (rPCR) has been demonstrated as a more rapid and sensitive method of VTEC detection, compared with enzyme immunoassay and culture (*7*).

**Figure:**
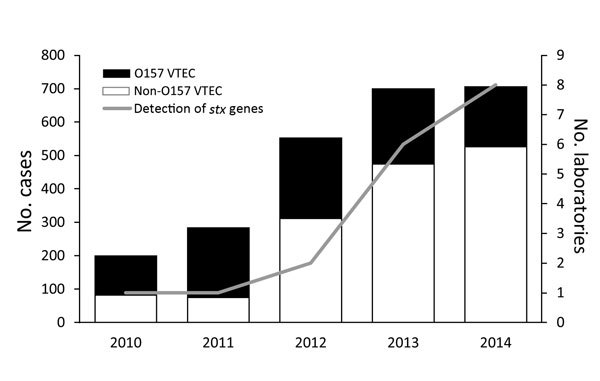
Number of reported cases of O157 and non-O157 VTEC infection and number of laboratories performing PCR-based detection of *stx* gene, by year, Ireland, 2010–2014. *stx*, Shiga toxin; VTEC, verotoxigenic *Escherichia coli*.

We conducted a survey of clinical microbiology laboratories in Ireland during 2014 to assess laboratory practices for the detection and confirmation of VTEC infection. The survey included 45 questions, arranged in 4 categories: laboratory details (e.g., contact details, address, and public/private status); sample type, selection, and requirements for VTEC testing; current methods (i.e., utilization and description of enrichment, culture, biochemical characterization, antimicrobial susceptibility testing, serotyping, molecular testing, and verotoxin detection methods), past changes to methods, and intended future changes to methods for VTEC analyses; and referral and reporting procedures. Ireland is home to 25 clinical microbiology laboratories, all of which were represented in the survey findings presented here. Laboratories included the VTEC National Reference Laboratory in Cherry Orchard Hospital, Dublin, which provides the referral service for this pathogen to all clinical laboratories in Ireland, whether public or private. Laboratories were categorized into 3 groups based on their testing strategy: culture-based detection, PCR-based detection, or both. An analysis of the introduction of molecular-based detection of VTEC infection (i.e., rPCR of verotoxin genes *vt*1 and *vt*2) in clinical laboratories in Ireland indicated a timeline for the implementation of these methods from 2010 to 2014. Before 2012, the reference laboratory was the sole laboratory implementing PCR-based detection of VTEC. In 2012, a regional clinical laboratory introduced an automated molecular platform for the detection of VTEC and other gastrointestinal pathogens. Four more laboratories introduced this molecular platform in 2013, with another 2 converting in 2014. Five clinical laboratories expressed an intention to introduce PCR-based detection of VTEC starting in 2015.

These data represent a shifting trend from the conventional culture-based detection of fecal coliforms (with confirmatory testing as VTEC), to a direct molecular approach in which stool specimens are tested initially for the presence of the verotoxin genes. This move toward molecular detection of VTEC infection is consistent with the increased number of non-O157 VTEC cases reported in Ireland in recent years (Figure). Similar results were reported in a study of the same nature conducted in Washington, USA (*8*). Along with the increased number of clinical laboratories participating in molecular detection of VTEC, a concomitant shift has occurred in the culture-based detection of VTEC. The introduction of an agar for the detection of Shiga-toxin–producing *E. coli* (CHROMagar STEC; Mast Diagnostika, Reinfeld, Germany) to laboratories, which is selective for the top 6 non-O157 serogroups (O26, O45, O103, O111, O121, and O145), supports the recently held view that a range of VTEC serogroups are now considered a major cause of human VTEC infection (*5*,*9*).

With the exception of the reference laboratory, molecular VTEC detection in clinical laboratories in Ireland use a commercial rPCR-based platform. This method, which selects for verotoxin genes, accelerates more sensitive detection compared with traditional culture-based methods.

Our own recent observations in Ireland (*10*,*11*) may very well represent the start of a global trend toward molecular detection of verotoxin genes from clinical and other samples, to replace culture-based methods, as a first step in the detection of VTEC (*12*). Our findings suggest that increases in notifications of VTEC infections, and particularly those caused by non-O157 serotypes, be interpreted in light of this paradigm shift in infectious disease diagnostic methods.
